# Adult-Onset Asthma With Periocular Xanthogranuloma (AAPOX) Associated With IgG4-Related Disorder: A Case Report and Review of Current Literature

**DOI:** 10.7759/cureus.82617

**Published:** 2025-04-20

**Authors:** Anil K Sahu, Saumya R Tripathy, Manoj K Parida, Sarit S Patnaik, Bidyut K Das

**Affiliations:** 1 Rheumatology, Srirama Chandra Bhanja Medical College and Hospital (SCBMCH), Cuttack, IND

**Keywords:** adult-onset asthma with periocular xanthogranuloma (aapox), igg4-related disorder (igg4-rd), igg4-related ophthalmic disease, periorbital swelling, rituximab

## Abstract

Adult-onset asthma with periocular xanthogranuloma (AAPOX) is a rare orbital xanthogranulomatous disease characterized by periocular yellow to orange cutaneous papules and nodules, usually associated with lymphadenopathy and adult-onset asthma. Immunoglobulin G4-related disease (IgG4-RD) is a systemic, immune-mediated condition of unknown etiology characterized by distinct histopathological features. Here, we report the rare case of a 57-year-old asthmatic woman who developed AAPOX concurrently with IgG4-RD, showing a favorable response to rituximab, along with a review of current literature. She presented with bilateral periorbital violaceous nodules and bilateral parotid swellings that had been present for the past seven years. Histopathological examination of the lower eyelid revealed Touton giant cells and foamy histiocytes, consistent with xanthogranuloma, while a biopsy from the left parotid swelling demonstrated features suggestive of IgG4-RD. A literature review using the keywords "IgG4RD", "AAPOX", "co-existence", and "association" identified 11 articles describing 17 cases with overlapping features. There was a male predominance, with a mean age of presentation of 49 years. Common ophthalmological findings included bilateral periorbital swelling with xanthelasma-like lesions. Systemic manifestations included asthma, involvement of the lacrimal and parotid glands, lymphadenopathy, and pancreatitis. Treatment with prednisone and rituximab proved effective in most reported cases. It remains uncertain whether these two entities represent a spectrum of a single disease or a nonspecific immunological overlap.

## Introduction

Adult-onset asthma with periocular xanthogranuloma (AAPOX) is a rare orbital xanthogranulomatous disease and a form of non-Langerhans cell histiocytosis, first described by Jakobiec et al. [1]. Although its exact etiology remains unclear, an allergic reaction is suspected to play a role. The presence of non-Langerhans histiocytes in the periorbital tissues suggests an overactive immune response involving the mononuclear phagocyte system. Clinically, AAPOX is characterized by periocular yellow to orange cutaneous papules and nodules, usually associated with lymphadenopathy and adult-onset asthma. It shows a male predominance and affects adults between 27 and 74 years of age [1,2].

Immunoglobulin G4-related disease (IgG4-RD) is a multisystemic immune-mediated disease of unknown etiology, characterized by inflammation and tissue fibrosis. Specific histopathological features include lymphoplasmacytic infiltration with a predominance of IgG4-positive plasma cells, storiform fibrosis, and obliterative phlebitis [3]. First described in 2001 in a patient with autoimmune pancreatitis [4], IgG4-RD usually affects individuals between the fifth and seventh decades of life, with a mean age of 50 years [5]. Its male preponderance contrasts with that of most classic autoimmune conditions. It can virtually affect any organ system, most commonly the salivary glands, orbit (periorbital areas), and retroperitoneum. It can mimic a range of other conditions, including autoimmune disorders, infections, and malignancies. Pathogenesis is thought to involve multiple mechanisms, including genetic predisposition and activation of both innate and adaptive immune pathways. Although elevated serum IgG4 levels are a characteristic finding, their role in the disease’s pathogenesis remains uncertain.

AAPOX and IgG4-RD share overlapping clinical, pathological, and serological features [6,7]. Both conditions are commonly treated with corticosteroids. Rituximab, a chimeric monoclonal antibody targeting CD20-positive B cells, has shown promising results in IgG4-RD [8-10]. We describe a patient diagnosed with both IgG4-RD and AAPOX who was intolerant to steroids but responded well to rituximab. Additionally, we present a review of the current literature exploring the relationship between these two conditions.

## Case presentation

A 57-year-old teacher presented with a seven-year history of painless, gradually progressive bilateral periorbital swelling. She had previously been treated with oral steroids and other disease-modifying anti-rheumatic drugs, including methotrexate, with minimal clinical improvement. The steroids were discontinued after she developed glaucoma. She also had a six-year history of bronchial asthma, managed with metered-dose inhalers containing inhaled steroids and long-acting beta-agonists.

**Figure 1:**
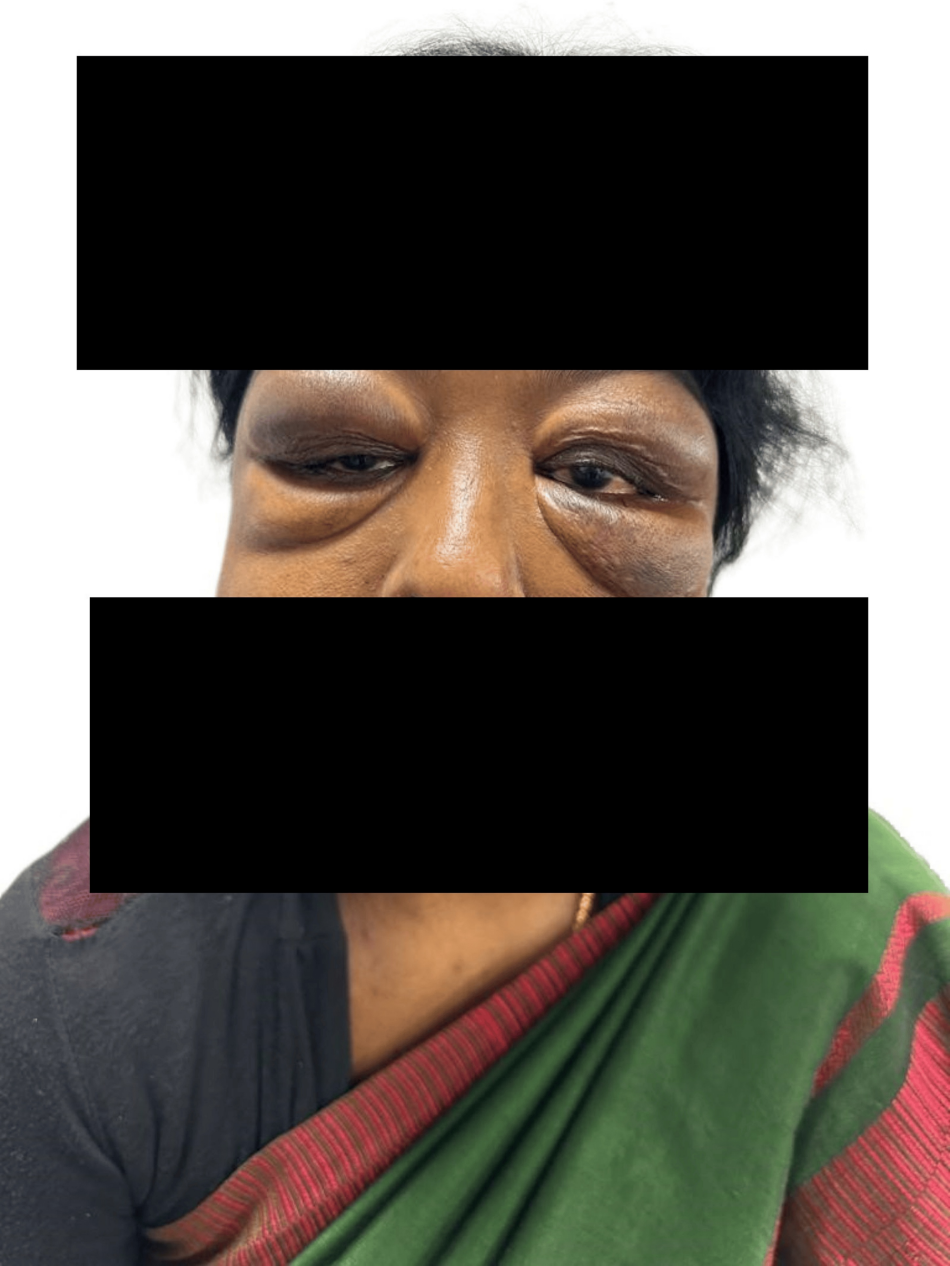
Patient characteristics before treatment, showing bilateral swelling of the eyelids Note: Written informed consent to include this image in an open-access article was obtained from the patient.

On examination, there were bilateral, nodular, firm, and non-tender swellings over the eyelids (Figure 1). The major salivary glands, including the submandibular and parotid glands, were enlarged, with multiple firm nodules palpable on the surface. Ocular examination revealed a visual acuity of 20/20 in both eyes, with intra-ocular pressures of 17 mmHg in the right eye and 18 mmHg in the left eye. There were no symptoms or signs of ocular sicca. Systemic examination was unremarkable. Routine blood tests showed elevated acute-phase reactants, increased serum total protein with reversal of the albumin-to-globulin ratio, and raised serum IgG4 levels (Table 1).

**Table 1:** Relevant blood tests

Laboratory study	Test values	Reference range
Hemoglobin	10 g/dL	11-17
Erythrocyte sedimentation range (ESR)	45 mm/hour	0-20
C-reactive protein (CRP)	16 mg/dL	<6
Serum total protein	8.5 g/dL	6-8
Serum albumin	3.1 g/dL	3.5-5.5
Serum globulin	5.4 g/dL	2-3.5
Serum IgG4 levels	3500 mg/dL	<135

Other investigations, including renal and liver function tests, serum calcium, serum angiotensin-converting enzyme (ACE) levels, and anti-nuclear antibody (ANA), were within normal limits. Serum protein electrophoresis and bone marrow examination ruled out plasma cell dyscrasia. A CT scan of the abdomen showed multiple sub-centimetric abdominal lymph nodes, along with evidence of retroperitoneal fibrosis.

**Figure 2:**
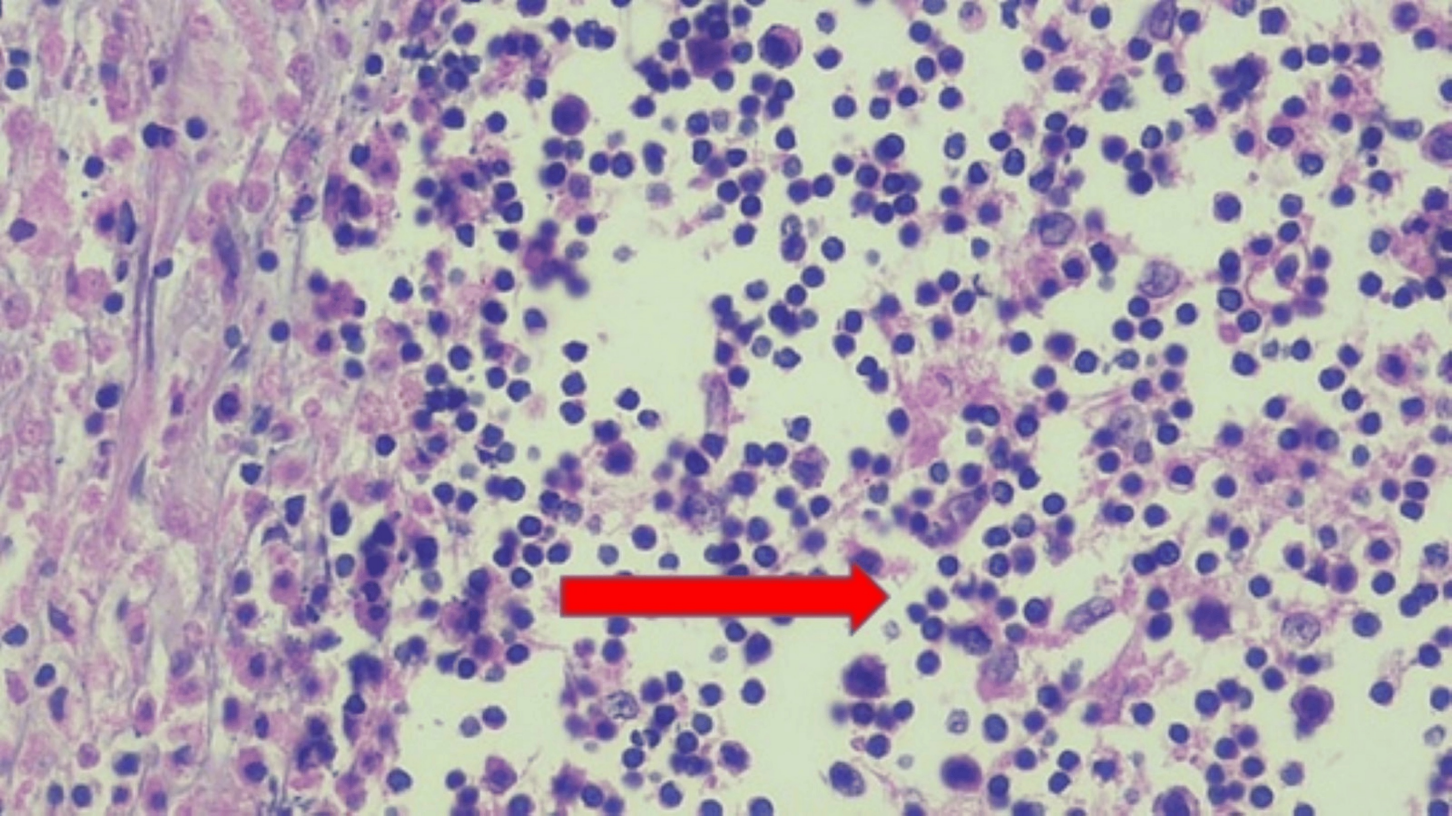
Biopsy of the left lower eyelid showing Touton giant cells (arrow) with foamy histiocytes

**Figure 3:**
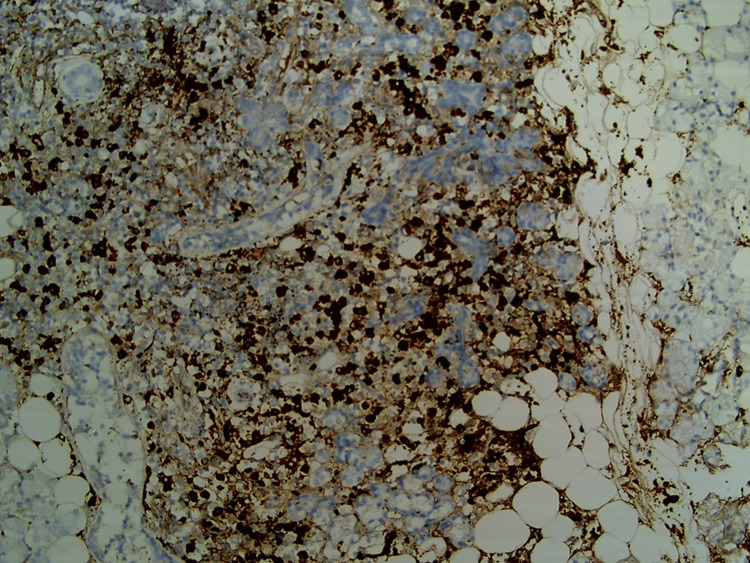
Biopsy of the left parotid gland showing an abundance of IgG4-positive plasma cells

Biopsy of the left lower eyelid revealed Touton giant cells with foamy histiocytes (Figure 2), which were positive for CD68 and CD163 and negative for S100 and CD1a, findings consistent with xanthogranuloma. Genomic screening for histiocytic disorders, including mutations in MAPK, BRAF, NRAS, KRAS, PIK3CA, and ALK gene, was negative. Positron emission tomography (18FDG-PET-CT) demonstrated increased uptake in the bilateral lacrimal, parotid, and submandibular glands, along with generalized lymphadenopathy involving axillary, mediastinal, and pelvic lymph node groups. Given the parotid swelling and elevated serum IgG4 levels, a biopsy of the left parotid gland was performed, which revealed peri-ductal and peri-lobular fibrosis, lymphoplasmacytic infiltrates, and an abundance of IgG4-positive plasma cells (60-70/hpf), suggestive of IgG4-RD (Figure 3).

**Figure 4:**
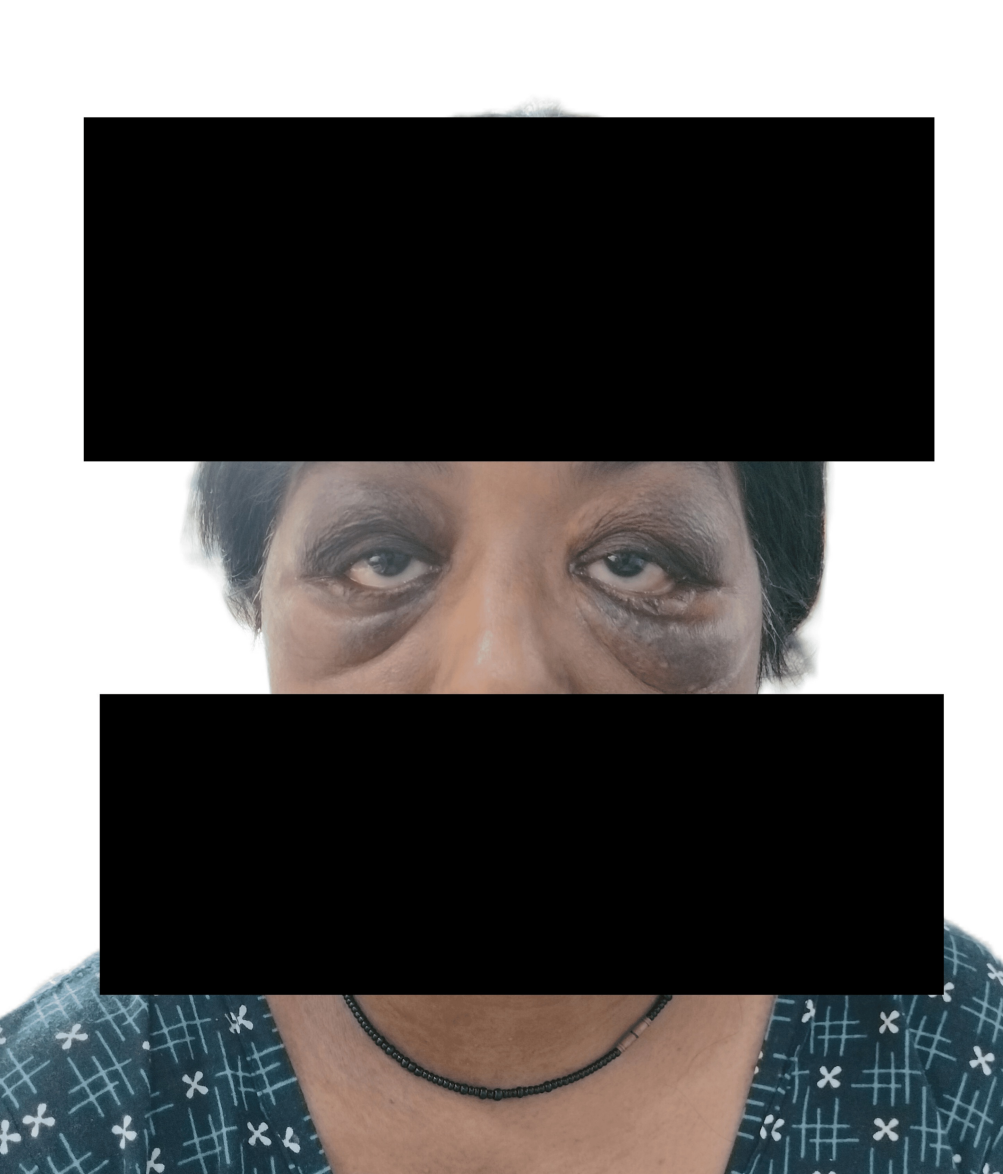
Patient characteristics after treatment, showing significant reduction of the periorbital swelling Note: Written informed consent to include this image in an open-access article was obtained from the patient.

Based on the clinical and histological findings, a diagnosis of AAPOX with overlapping IgG4-RD was made. The patient received an induction cycle of rituximab (1000 mg), administered in two doses at an interval of two weeks. This resulted in a significant reduction of the periorbital swelling (Figure 4).

## Discussion

Search strategy

Five databases (PubMed, Scopus, Embase, Science Direct, and Ovid) were screened for English-language articles published from database inception to January 15, 2025, using the keywords "IgG4RD", "AAPOX", "co-existence", and "association". The search query used was “AAPOX AND IgG4 related disorder AND (association OR coexistence)”. We included case reports, case series, letters, and original research articles that discussed both IgG4-RD and AAPOX. Only articles published in English were considered. Duplicate articles and abstracts were excluded from the review. We also included articles cross-referenced from the above articles. A flowchart detailing the article selection process, in accordance with the PRISMA guidelines for systematic reviews, is presented in Figure 2.

**Figure 5:**
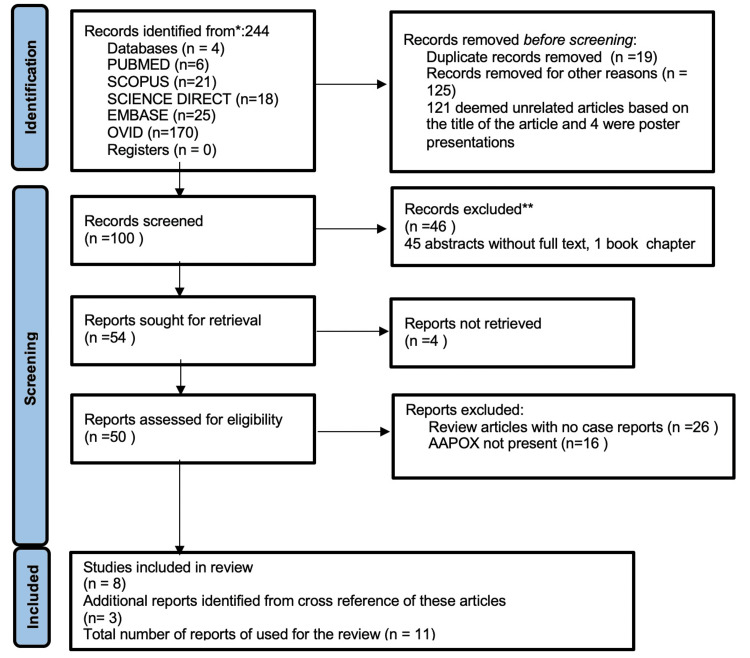
Flowchart of the article selection process for this systematic review

We identified 11 articles describing 17 cases with co-existing AAPOX and IgG4-RD. The characteristics of these cases, along with our current case, are summarized in Table 2. Among these, 10 were male and 7 were female cases, with a male-to-female ratio of 1.4:1 [6-16]. Periorbital swelling was the most frequently reported manifestation, followed by systemic features such as lymphadenopathy, asthma, involvement of the lacrimal and parotid glands, and sclerosing pancreatitis. Among the treatments described, only corticosteroids and rituximab were consistently effective; other therapeutic options showed limited or no benefit.

**Table 2:** Characteristics of cases with AAPOX and IgG4-RD MP: methylprednisolone, PD: prednisolone, RTX: rituximab, AZA: azathioprine, MTX: methotrexate, CYS: cyclosporine.

Author(s) of the study	Age	Gender	Ophthalmic manifestation	Extraocular manifestation	Treatment
This study	57	F	B/L periorbital swelling	Adult-onset asthma	MP 100 mg (two cycles), RTX 1 g (two cycles)
Barke et al. [6]	58	F	B/L yellow-orange xanthelasma-like plaques, dry eye, and blepharoptosis	Adult-onset asthma, raised serum IgG4 levels	Intralesional triamcinolone, right orbitotomy, oral PD, RTX
Chhabra et al. [7]	52	F	Periorbital xanthelasmas	Mucoepidermoid carcinoma (MEC) of a minor salivary gland, pancreatitis, adult-onset asthma, lymphadenopathy, rhinosinusitis	Resection of MEC, oral PD, and MTX
Asproudis et al. [8]	36	M	B/L yellow upper and lower eyelid swelling	Adult-onset asthma, left parotid gland and submandibular lymph node enlargement	MP, PD 40 mg, RTX 1 g (two cycles)
Leung et al. [9]	61	M	B/L swollen eyelids with yellowish lesions	Autoimmune pancreatitis	PD, AZA, RTX
Pomponio et al. [10]	50	F	Yellow eyelid plaque	Salivary and lacrimal gland enlargement, lymphadenopathy, pulmonary mass	RTX
Burris et al. [11]	57	M	Bilateral yellow upper and lower eyelid swelling	Adult-onset asthma, sclerosing pancreatitis	MP 100 mg IV, RTX 1 g (two cycles)
Honda et al. [12]	47	F	Bilateral upper and lower eyelid enlargement	Lacrimal gland enlargement, cervical lymphadenopathy	PD
London et al. [13]	65	M	Bilateral yellow upper and lower eyelid swelling	Adult-onset asthma, chronic sinusitis, cervical lymphadenopathy, left cheek swelling	PD
London et al. [13]	52	M	Bilateral orange upper and lower eyelid swelling	Adult-onset asthma, allergic sinusitis, obstructive sleep apnea	PD and MTX
London et al. [13]	33	F	Yellow swelling of the left upper eyelid	Adult-onset asthma, allergic sinusitis, bilateral axillary adenopathy	PD and MTX
Kubota et al. [14]	38	M	Bilateral swollen eyelids	Autoimmune pancreatitis, asthma	PD
Kubota et al. [14]	41	M	Bilateral swollen eyelids	Asthma cervical lymphadenopathy	PD
Kubota et al. [14]	48	F	Bilateral periocular mass	Asthma, ↑IgG4	PD
Kubota et al. [14]	34	F	Eyelid swelling	Asthma, ↑IgG4	PD 5 mg/day, CYS
Singh et al. [15]	67	M	Fullness of the right periorbital region	Sclerosing cholangitis	NA
Singh et al. [15]	36	M	Orbital lesions	Adult-onset asthma, upper respiratory and sinus problems	NA
Roggin et al. [16]	61	M	Bilateral yellow upper and lower eyelid swelling	Adult-onset asthma, nasal polyps, sclerosing pancreatitis, cervical lymphadenopathy	PD

We report a rare case of combined AAPOX and IgG4-RD disorder, which was successfully treated with rituximab. Adult orbital xanthogranulomatous disease (AOXGD) refers to a group of diverse orbital and ocular adnexal disorders, classified under type II non-Langerhans histiocytosis. AOXGD is categorized into four types based on the extent and nature of involvement: adult-onset xanthogranuloma (AOX), AAPOX, Erdheim-Chester disease (ECD), and necrobiotic xanthogranuloma (NBX) [2]. AAPOX is a relatively rare condition, with fewer than 50 cases reported in the literature to date [17]. In addition to the periorbital swelling, patients may also present with proptosis and restricted movement of the extraocular muscles. Diplopia, though less common, can occur when extraocular muscle involvement is significant. Consistent with most documented cases, our patient exhibited a gradual onset of bilateral periorbital swelling. However, a notable difference was the absence of keratoconjunctivitis sicca symptoms, commonly reported in similar cases, highlighting the variability in clinical presentation.

The most common extraocular association of AAPOX is bronchial asthma, which typically follows the onset of orbital lesions by a few months to several years, as observed in our index patient. It is also frequently associated with lymphadenopathy, paraproteinemia, and, more rarely, lymphoproliferative disorders such as multiple myeloma, chronic lymphocytic leukemia, and non-Hodgkin’s lymphoma. In many cases, AAPOX occurs in conjunction with systemic IgG4-RD, often accompanied by elevated serum IgG4 levels. Additionally, it is commonly linked with chronic rhinosinusitis, nasal polyps, elevated serum immunoglobulin E (IgE) levels, and eosinophilic infiltration in the affected tissues [2].

The ophthalmic manifestations of IgG4-RD have been documented in numerous studies, involving structures such as the lacrimal glands and ducts, extraocular muscles, orbital soft tissue, and sclera. These manifestations are now collectively referred to as IgG4-related ophthalmic disease (IgG4-ROD). The most common presentation is dacryoadenitis. In rare cases, IgG4-ROD may involve the orbital apex or orbital nerves. Proptosis can also occur, typically due to enlargement of the extraocular muscles, but may also result from orbital pseudo-tumors.

Common histopathologic findings in AAPOX include sheets of mononucleated foamy histiocytes accompanied by lymphoplasmacytic infiltration and Touton giant cells. Immunohistochemical analysis revealed strong expression of CD68, CD163, and factor XIIIa in foamy histiocytes, while they are negative for CD21, CD35, S100, and CD1a [2]. 

Furthermore, the typical histopathological features of IgG4-RD include dense lymphoplasmacytic infiltrates with a predominance of IgG4+ plasma cells, obliterative phlebitis, and storiform fibrosis. Immunohistochemically, a significantly elevated IgG4+/IgG+ plasma cell ratio, often exceeding 40%, is a strong indicator of IgG4-RD [18]. However, the absolute number of plasma cells observed per high-powered field can vary depending on the organ involved. While elevated serum IgG4 levels are a distinct feature of the disease, they are not exclusive to IgG4-RD and may also be observed in conditions such as granulomatosis with polyangiitis, eosinophilic granulomatosis with polyangiitis, bronchiectasis, biliary diseases (e.g., primary sclerosing cholangitis), pancreatic cancer, and others. Conversely, serum IgG4 levels can be normal in biopsy-proven cases, underscoring that this parameter alone cannot be relied upon for diagnosis [19]. As in AAPOX, FDG-PET scanning in IgG4-RD is valuable for assessing the extent of systemic involvement and monitoring treatment response. In our case, although storiform fibrosis and obliterative phlebitis were absent, consistent with many previously reported cases, the diagnosis of IgG4-RD was supported by typical organ involvement, markedly elevated serum IgG4 levels, and the presence of IgG4-predominant plasma cells.

For the management of AAPOX, treatment options include local and systemic steroids, immunosuppressive agents, and debulking surgery. Intralesional corticosteroids have shown efficacy in a few patients; however, most patients require oral steroids, typically initiated at a starting dose of 1 mg/kg/day with gradual tapering. Despite initial responses, recurrences are common upon tapering. Several case reports have described the use of immunosuppressive agents, most commonly methotrexate, followed by azathioprine, cyclosporine, and cyclophosphamide, with varying degrees of success. Rituximab has demonstrated durable responses, particularly in refractory cases. Surgical debulking should be considered only as a last resort.

IgG4-RD responds remarkably well to corticosteroids, especially when treatment is initiated early in the disease course. A commonly adopted regimen begins with prednisolone at a daily dose of 0.6 to 1.0 mg per kg of body weight. After 2-4 weeks, the dose is tapered gradually by 5 mg every 1-2 weeks, as per clinical response. Although immunosuppressive agents such as methotrexate, azathioprine, and mycophenolate mofetil have been used, there is limited robust evidence supporting their efficacy. Similar to AAPOX, rituximab has demonstrated substantial effectiveness in IgG4-RD. Additionally, recent clinical trials have shown promising results with inebilizumab, an anti-CD19 monoclonal antibody, in reducing disease flares in IgG4-RD [20].

Our patient initially demonstrated a transient response to oral prednisolone at a dosage of 10 mg daily. However, it was discontinued due to the development of elevated intraocular pressure (24 mmHg in the right eye and 26 mmHg in the left eye), accompanied by ocular pain. Subsequently, she was put on methotrexate, cyclosporine, and azathioprine, but showed no significant symptomatic improvement. The patient was then administered two doses of rituximab (1000 mg each), resulting in a substantial regression in periorbital swelling. Follow-up testing revealed a downward trend in serum IgG4 levels. She has remained clinically stable with no signs of disease activity during the six-month period following rituximab therapy.

## Conclusions

AAPOX and IgG4-RD share certain clinical manifestations, pathological features, and treatment responses. However, the exact nature of their relationship remains unclear. It is uncertain whether these two diseases represent a continuum of a common spectrum disorder or a nonspecific immunological response. Further study is necessary to establish a definite link between AAPOX and IgG4-RD.
